# Supplementary material for: Ancient Mitogenomes Suggest Stable Mitochondrial Clades of the Siberian Roe Deer
Source: Genes (Basel). 2022 Jan 8;13(1):114. doi: 10.3390/genes13010114 (PMC8774404; doi:10.3390/genes13010114)
Supplement: Supplementary file 1 [file genes-13-00114-s001.zip › genes-1467022-Supporting Information.pdf]

Supporting Information

Figure S1: Photos of four roe deer sub-fossil specimens. (a): CADG573, (b): CADG580, (c): CADG626, (d): CADG842.

Figure S2: Radiocarbon dating of four roe deer sub-fossil specimens. Lab Number: Beta-561491 (CADG573), Beta-561492 (CADG580), Beta-560182 (CADG626) , Beta-604140 (CADG842).

Figure S3: Coverage plots of the mitochondrial genomes obtained in this study.

Figure S4: Mitochondrial read length distribution of four roe deer sub-fossil specimens.

Figure S5: Mitochondrial DNA damage plots for four roe deer sub-fossil specimens. *X axis* represents position from 5' (left) and 3' (right) read ends. Red line corresponds to C to T substitutions and blue line corresponds to G to A substitutions.

Figure S6: Maximum-likelihood phylogenetic tree of *Capreolus* based on complete mitochondrial genomes. *A. alces* was selected as outgroup. Names of four Chinese samples are shown in red. Node label represents the bootstrap value.

Table S1: Complete mitochondrial genomes used in this study.

Table S2: Siberian roe deer sample information for each location per region.

Table S3: Substitution models for different mitochondrial DNA partitions determined using Partitionfinder 2.

Table S4: Information of the specimens and mitochondrial genomes obtained in this study.

Table S5: Average node ages from BEAST (Ma).

Table S6: Mapping values of four Northeastern China roe deer.

Figure S1: Photos of four roe deer sub-fossil specimens. (a): CADG573, (b): CADG580, (c): CADG626, (d): CADG842.

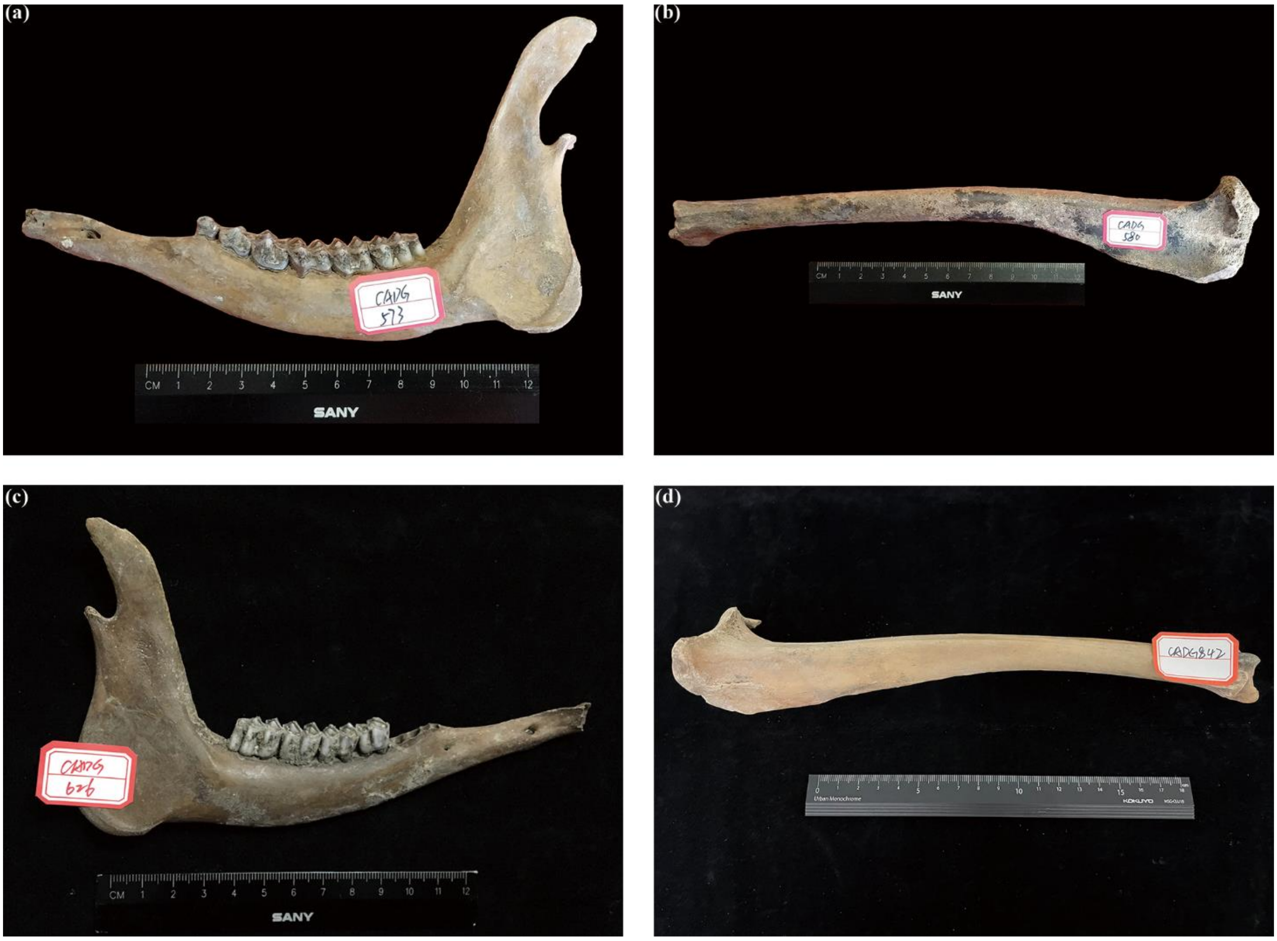

Figure S2: Radiocarbon dating of four roe deer sub-fossil specimens. Lab Number: Beta-561491 (CADG573), Beta-561492 (CADG580), Beta-560182 (CADG626) , Beta-604140 (CADG842).

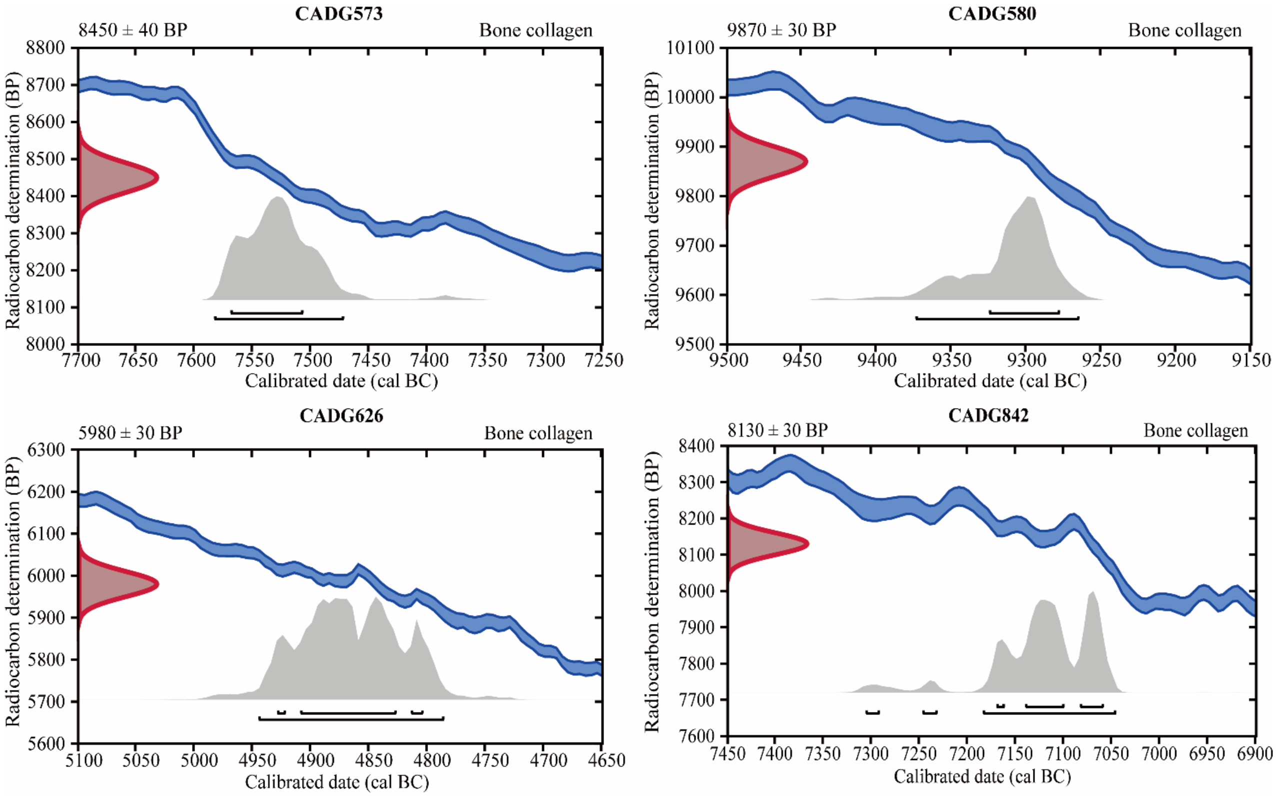

Figure S3: Coverage plots of the mitochondrial genomes obtained in this study.

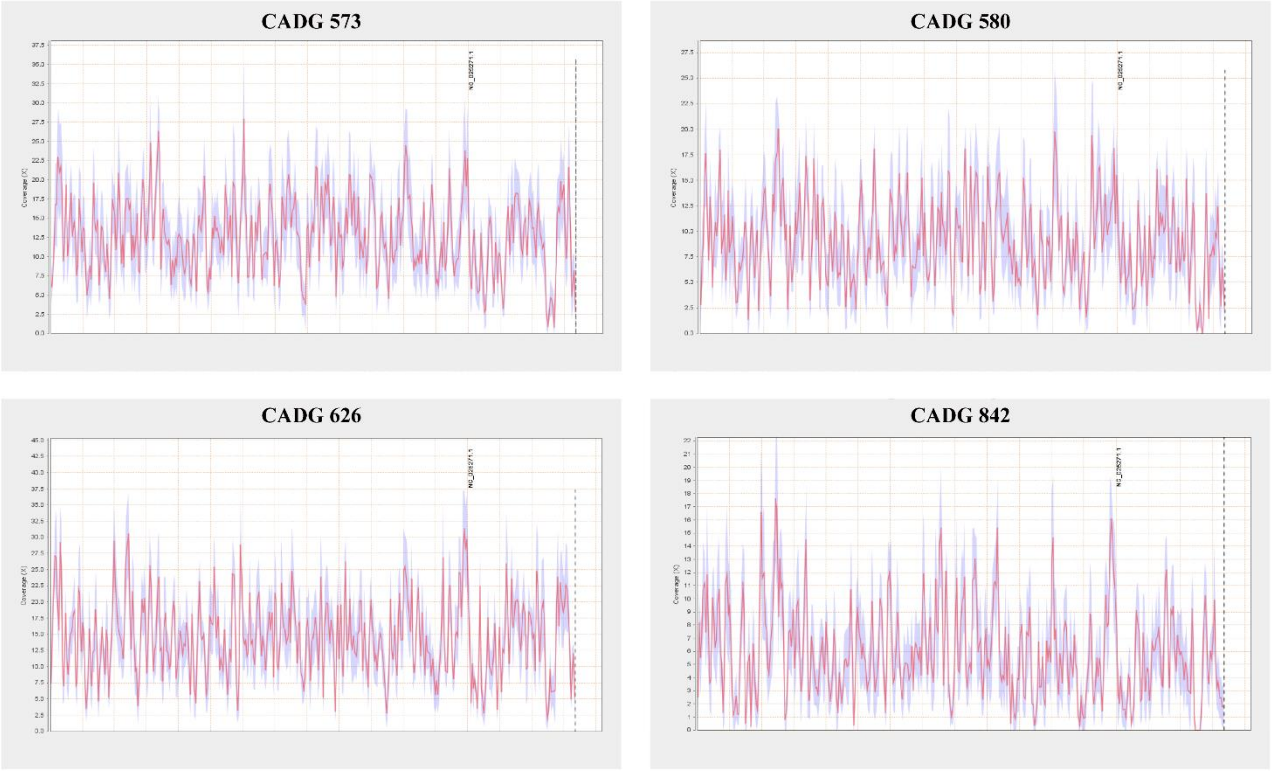

Figure S4: Mitochondrial read length distribution of four roe deer sub-fossil specimens.

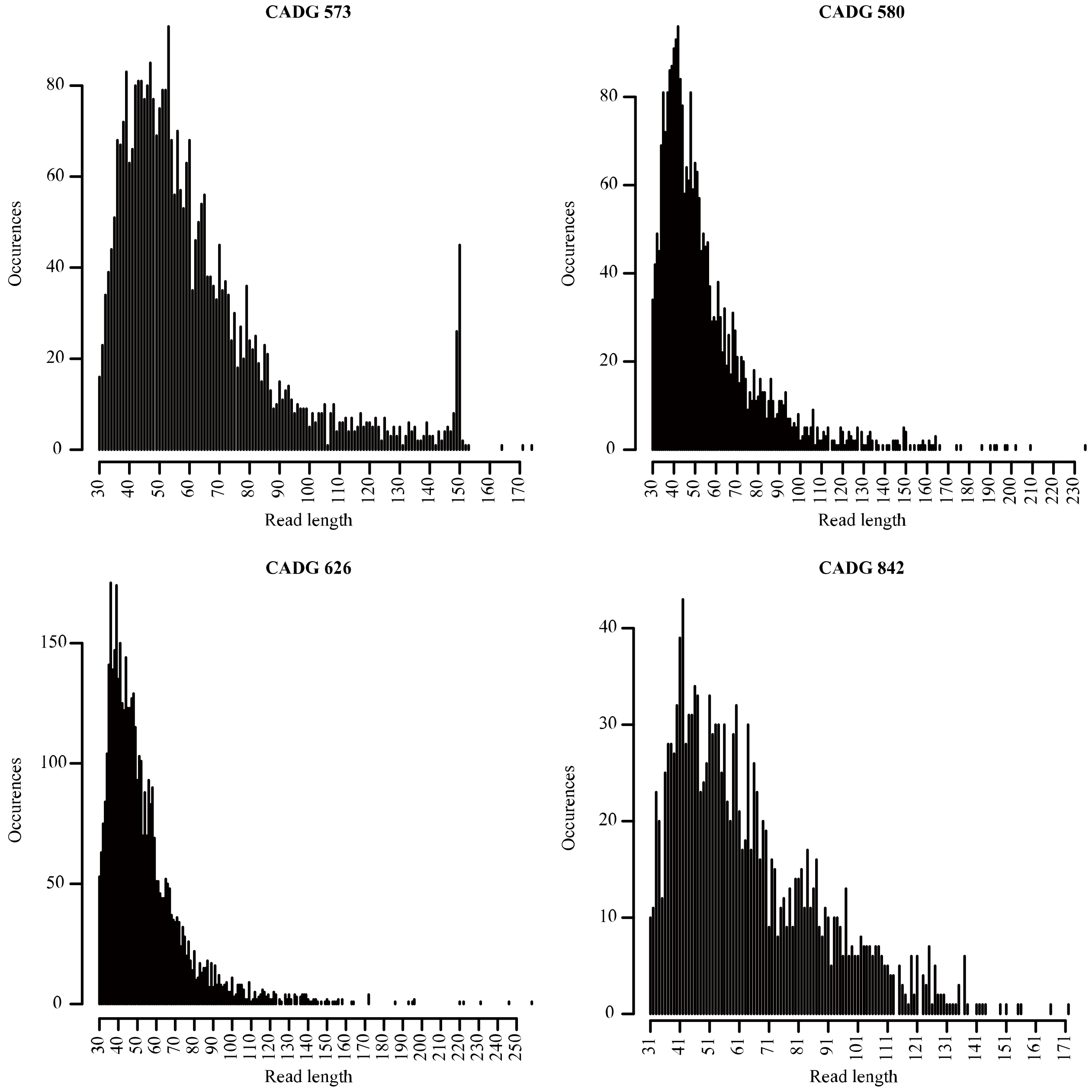

Figure S5: Mitochondrial DNA damage plots for four roe deer sub-fossil specimens. *X axis* represents position from 5’ (left) and 3’ (right) read ends. Red line corresponds to C to T substitutions and blue line corresponds to G to A substitutions.

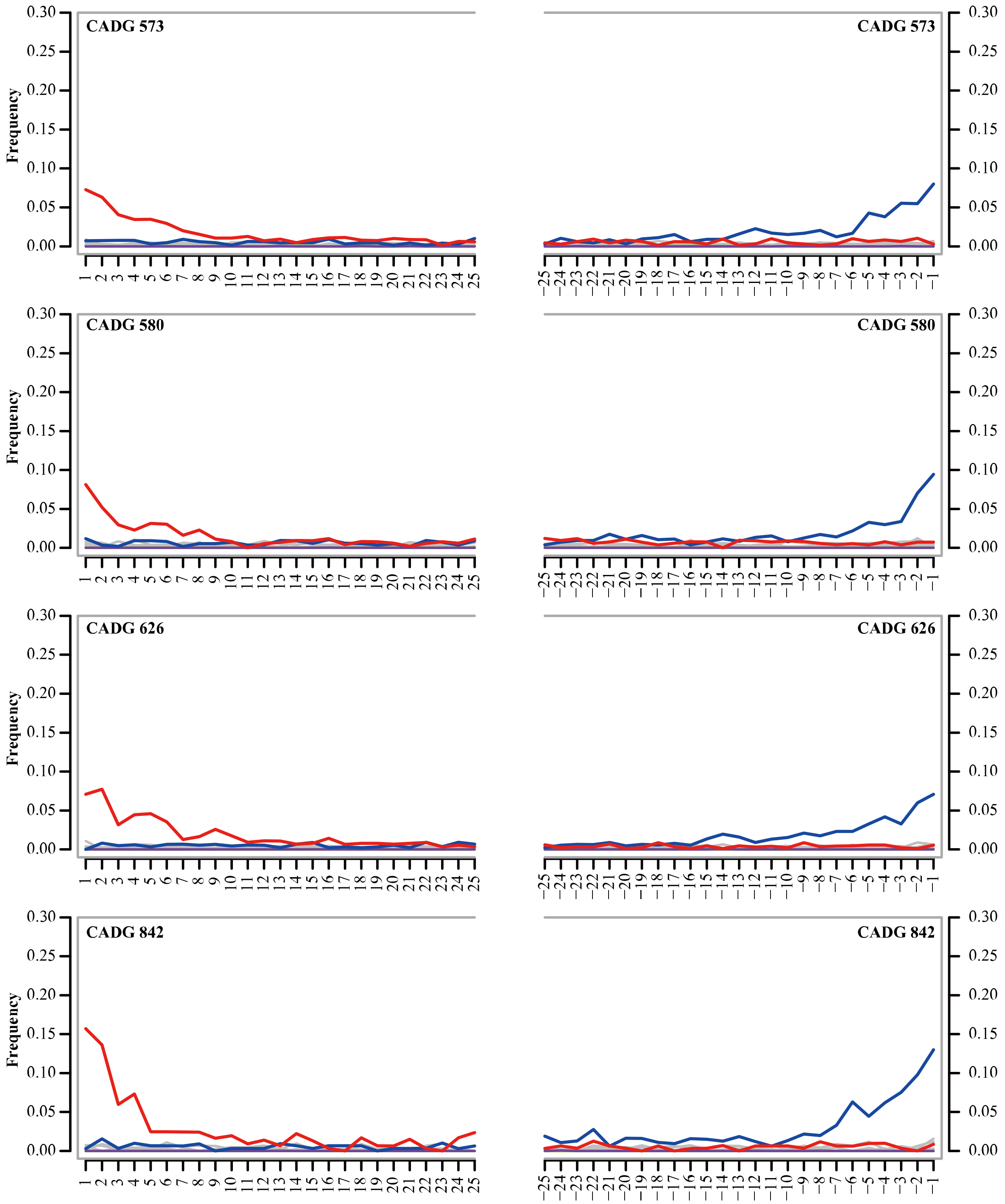

Figure S6: Maximum-likelihood phylogenetic tree of *Capreolus* based on complete mitochondrial genomes. *A. alces* was selected as outgroup. Names of four Chinese samples are shown in red. Node label represents the bootstrap value.

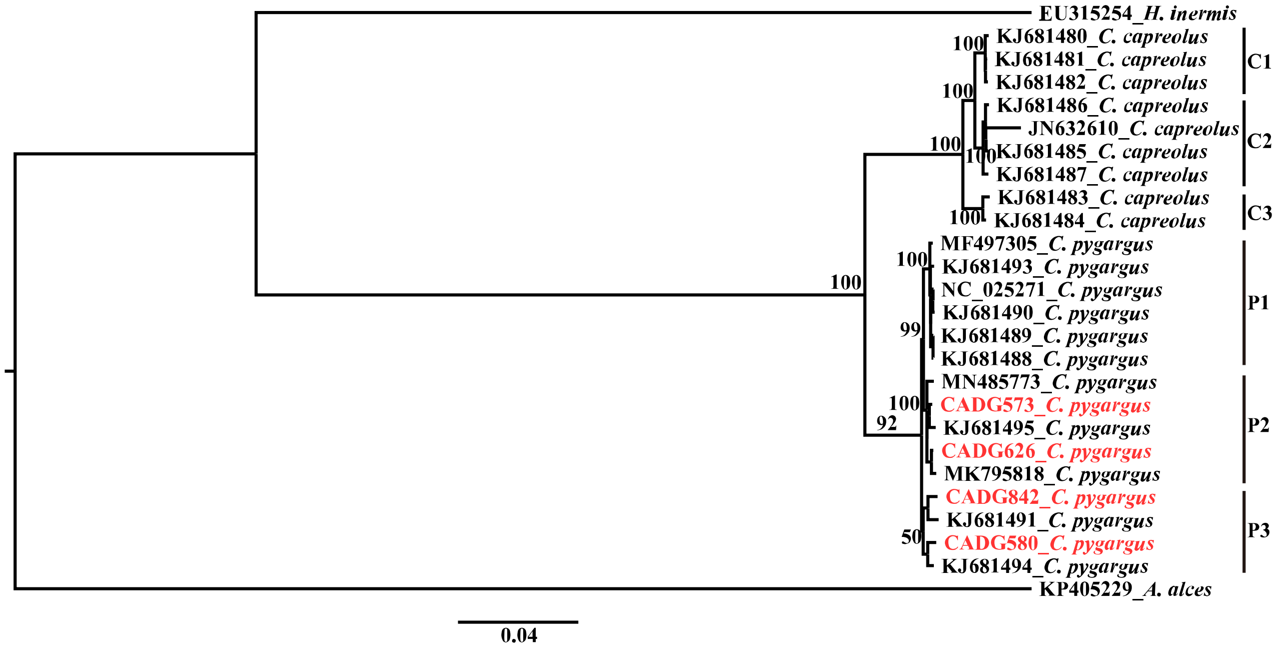

Table S1: Complete mitochondrial genomes used in this study.

GenBank	Species-specific mtDNA lineage	Location	Age (Ybp)
NC_025271	*Capreolus pygargus*	Kurgan, Russia	modern
KJ681493	*Capreolus pygargus*	Kurgan, Russia	modern
MF497305	*Capreolus pygargus*	Wutaishan, Korea	modern
KJ681495	*Capreolus pygargus*	Luchegorsk, Russia	modern
KJ681494	*Capreolus pygargus*	Luchegorsk, Russia	modern
MK795818	*Capreolus pygargus*	Shanxi, China	modern
MN485773	*Capreolus pygargus*	Harbin, Heilongjiang, China	modern
KJ681490	*Capreolus pygargus*	Przemyśl, Poland	modern
KJ681488	*Capreolus pygargus*	Pasłęk, Poland	modern
KJ681489	*Capreolus pygargus*	Bielsk Podlaski, Poland	modern
KJ681491	*Capreolus pygargus*	Wola Niżna, Poland	modern
KJ681480	*Capreolus capreolus*	Pasłęk, Poland	modern
KJ681481	*Capreolus capreolus*	Białobrzegi, Poland	modern
KJ681482	*Capreolus capreolus*	Banie, Poland	modern
KJ681483	*Capreolus capreolus*	Lubaczów, Poland	modern
KJ681484	*Capreolus capreolus*	Lubaczów, Poland	modern
KJ681485	*Capreolus capreolus*	Lublin, Poland	modern
KJ681486	*Capreolus capreolus*	Przemyśl, Poland	modern
KJ681487	*Capreolus capreolus*	Jarosław, Poland	modern
JN632610	*Capreolus capreolus*	Paris, France	modern
EU315254	*Hydropotes inermis*	China	modern
KP405229	*Alces alces cameloides*	Inner Mongolia, China	modern

Table S2: Siberian roe deer sample information for each location per region.

Region	Location	Sequence
SKJ	South Korea, Jeju	Hap80, Hap81, Hap82, Hap83, Hap84, Hap85, Hap86 Hap87, Hap88
SKM	South Korea, mainland	Hap17, Hap19, Hap20, Hap33, Hap34, Hap45, Hap49, Hap50, Hap51, Hap52, Hap53, Hap54, Hap55, Hap56, Hap58, Hap59, Hap60, Hap95, Hap108, MF497305
RPRA	Russia, PrimorskyKrai	Hap1, Hap5, Hap6, Hap7, Hap18, Hap21, Hap24, Hap25,Hap26, Hap28, Hap36, Hap37, Hap38, Hap39, Hap42, Hap43, Hap46, Hap48, Hap57, Hap61, Hap62, Hap63, Hap65, Hap66, Hap67, Hap68, Hap69, Hap70, Hap71, Hap90, Hap92, Hap93, Hap94, Hap96, Hap97
	Russia, Amur region	Hap3, Hap9, Hap22, Hap31, Hap44, Hap47, Hap91, Hap104, Hap105, KJ681495, KJ681494, 685, 692, 754, 1156
	Heilongjiang, China	CADG573, CADG580, CADG626, CADG842, MN485773
RYA	Russia, Yakutia	Hap10, Hap11, Hap29, Hap30, Hap89, Hap101, Hap102, Hap107, 1160, 1168
RSMG	Russia, Sokhondinsky	Hap12, Hap13, Hap23, Hap40, Hap41, Hap64, Hap74, Hap98, Hap103, Hap106, Hap109, Hap110
	Mongolia, Northern part	Hap2, Hap4, Hap8, Hap14, Hap15, Hap16, Hap27, Hap32, Hap35, Hap72
	Russia, Buryatia	149, 150
RARN	Russia, Altay	Hap98, Hap112
	Russia, Novosibirsk	Hap111
	Kazakhstan	161
	Russia, Krasnoyarsk	334, 723
	Russia, Khakassia	721, 724
	Russia, Irkutsk	154, 767, 768
RUKO	Russia, Ural	Hap73, Hap75, Hap76, Hap79, Hap99, Hap100
	Russia, Kurgan	Hap73, Hap75, Hap77, Hap78, Hap79, Hap99, Hap100, NC_025271, KJ681493
	Russia, Orenburg	Hap75, Hap76, Hap77
	Russia, Sverdlovsk	314, 319
PLD	Poland	KJ681488, KJ681489, KJ681490, KJ681491

Table S3: Substitution models for different mitochondrial DNA partitions determined using Partitionfinder 2.

**Name**	**Best model**	**length (bp)**	**Genomic features**
1	GTR+I+G	5853	Gln, ND6_CP3, ND6_CP2, Ile, Leu2, Lys, Leu1, Ala, ND4L_CP1, ND4_CP1, Arg, His, ATP8_ATP6_CP3, _Val, ND2_CP1, Phe, Ser2, Gly, ND5_CP1, 12s, Thr, Ser1, Tyr, Asp, 16s, Glu
2	GTR+I	2196	COX1_CP1, Trp, Met, Cys, Pro, ND1_CP1, COX2_CP1, Asn, COX3_CP1, ND3_CP1, CYTB_CP1
3	GTR+I	3603	COX2_CP2, COX1_CP2, CYTB_CP2, ND1_CP2, ND4L_CP2, ATP8_ATP6_CP1, ND5_CP2, COX3_CP2, ND4_CP2, ND2_CP2, ND3_CP2
4	GTR+G	2981	COX3_CP3, ND4_CP3, ND5_CP3, CYTB_CP3, ND1_CP3, ND3_CP3, ND6_CP1, ND4L_CP3, COX2_CP3, ND2_CP3
5	GTR+I	795	COX1_CP3, ATP8_ATP6_CP2

[1] Alternative models specified for RAxML: GTR, GTR+G, GTR+I, GTR+I+G

Table S4: Information of the specimens and mitochondrial genomes obtained in this study.

Sample No.	Taxon	Skeletal element	Location	Radiocarbon age (^{14}C, BP)	Calibrated radiocarbon age (BP)	Aligned reads	Sequence length (bp)	Average read depth	Average fragment lengths
CADG573	*Capreolus pygargus*	mandible	Qinggang, Heilongjiang, China	8,450 ± 40	95.4% (9,533-9,422)	3,321	16,229	12.7	62.48
CADG580	*Capreolus pygargus*	tibia	Qinggang, Heilongjiang, China	98,70 ± 30	95.4% (11,324-11,215)	2,653	15,874	9.0	55.63
CADG626	*Capreolus pygargus*	mandible	Qinggang, Heilongjiang, China	68,95 ± 30	95.4% (6,895-6,736)	4,445	16,348	14.5	53.43
CADG842	*Capreolus pygargus*	tibia	Qinggang, Heilongjiang, China	8,130 ± 30	92.3% (9,134-8,996)	1,492	14,758	5.9	64.49

Table S5: Average node ages from BEAST (Ma).

Node	Average node ages	95% HPD
Alces alces cameloides + Hydropotes inermis + Capreolus	12.80	14.56 - 11.07
Hydropotes inermis + Capreolus	10.40	11.78 - 8.95
Capreolus	2.25	2.63 - 1.87
Capreolus pygargus	0.34	0.41 - 0.27
subclade P1	0.10	0.14 - 0.07
subclade P2	0.17	0.23 - 0.14
subclade P1 + subclade P2	0.27	0.33 - 0.21
subclade P3	0.30	0.37 - 0.24
Capreolus capreolus	0.68	0.81 - 0.55
subclade C1	0.08	0.11 - 0.05
subclade C2	0.32	0.39 - 0.24
subclade C1 + subclade C2	0.45	0.55 - 0.36
subclade C3	0.13	0.18 - 0.08

Table S6: Mapping values of four Northeastern China roe deer.

Sample No.	Library No.	Mapable reads	Mapped reads	Uniq mapped reads	Mapped bp	Endogenous content
CADG573	573-1	23621110	763	616	34362 bp	0.00002
	573-2	12940396	510	387	19603 bp	0.00002
	573-2-2	8004584	316	266	13040 bp	0.00003
	573-3	21466586	1072	898	61619 bp	0.00004
	573-3-2	26703903	1325	973	65933 bp	0.00003

	573-4	13524541	248	221	16313 bp	0.00001
CADG580	580-1	25393500	315	245	13800 bp	0
	580-2	5028856	71	59	2975 bp	0.00001
	580-2-1	16893357	224	181	9451 bp	0.00001
	580-3	20752176	718	576	31452 bp	0.00002
	580-3-1	22314574	779	563	31042 bp	0.00002
	580-4	13804631	445	362	16071 bp	0.00002
	580-4-1	12239573	425	334	14536 bp	0.00002
	580-4-2	7247154	238	215	9247 bp	0.00002
	580-5	10078748	241	214	12986 bp	0.00002
	580-6	10397188	190	171	9917 bp	0.00001
	580-7	12168949	118	102	8099 bp	0
	580-8	22243945	222	196	16410 bp	0
CADG626	626-1	23532713	181	145	8725 bp	0
	626-2	9445257	112	105	5208 bp	0.00001
	626-2-1	15426320	165	125	6388 bp	0
	626-2-2	13751167	128	111	5629 bp	0
	626-3	10284952	184	167	6739bp	0.00001
	626-3-1	23035930	491	380	15434 bp	0.00001
	626-4	13605408	304	269	11084 bp	0.00001
	626-4-1	25332315	647	520	21709 bp	0.00002
	626-5	22168508	709	602	32154 bp	0.00003
	626-6	27981931	922	811	48616 bp	0.00002
	626-7	15991152	748	587	34471 bp	0.00003
	626-8	28289412	1106	935	53364 bp	0.00003
CADG842	842-1	17290405	1697	1492	96218 bp	0.00008
